# Comparison of Surgical Outcomes between Canaloplasty and Schlemm's Canal Scaffold at 24 Months' Follow-Up

**DOI:** 10.1155/2016/3410469

**Published:** 2016-02-16

**Authors:** Stefano A. Gandolfi, Nicola Ungaro, Stella Ghirardini, Maria Grazia Tardini, Paolo Mora

**Affiliations:** Ophthalmology Unit, University Hospital of Parma, 43126 Parma, Italy

## Abstract

The results of canaloplasty (CP) and Hydrus Microstent (HM) implantation were retrospectively compared at 24 months' follow-up in a cohort of subjects referred to our Institution for uncontrolled IOP in primary or secondary (e.g., pseudoexfoliative and pigmentary) open-angle glaucoma. The outcome was labelled as “complete” success, “qualified” success, or “failure” if, two years after surgery, the eyes operated on needed “no” hypotensive medications, “some” hypotensive medications, or further glaucoma surgery to attain the target IOP, respectively. Both CP and HM implant allowed significant IOP reductions, with comparable rate of clinical success and safety profile. A slightly (albeit not significant) better trend for a “complete” clinical success was observed in the CP group.

## 1. Introduction

Creating an extraocular aqueous humour filtration is still the most popular surgical strategy in glaucoma worldwide. However, because of the well known bleb-related problems, a quest for new “bleb-free” procedures has been pursued for several years. For example, canaloplasty (CP) and the trabeculocanalicular devices implantable through a minimally invasive approach (the so called MIGS) are two surgical strategies not ending in external filtration.

CP consists of an “ab-externo” dilation of Schlemm's canal (SC) (via a transconjunctival and transscleral approach) obtained by intracanalicular injection of high molecular weight (HMW) viscoelastic and placement of a permanent intracanalicular tension suture [1]. MIGS devices are placed in the anterior chamber (AC) angle under gonioscopic view and via a clear cornea incision [2], sparing then the conjunctiva for later interventions. The Hydrus Microstent (Ivantis, Inc., Irvine, CA) is a MIGS device made of nitinol, (a nickel and titanium alloy widely used in ophthalmic and other medical applications [3]), currently approved for intraocular use in Europe. This device is an 8 mm long crescent-shaped open structure, curved to match the shape of SC. Once implanted, the microstent bypasses the trabecular meshwork and dilates SC over 3 clock hours to provide direct aqueous access from the AC to multiple collector channels [4, 5].

To date, some studies have compared the outcomes of CP with filtering surgery. The large majority of these reports showed that, in the short-to-mid term, both procedures are effective in achieving significant reduction of intraocular pressure (IOP), with a better efficacy profile for filtration surgery and a better safety profile for CP [6–9]. The hereby presented study was aimed to compare the 2-year clinical outcome of CP versus a bleb-free MIGS (i.e., the Hydrus Microstent (HM)), within the frame of a retrospective comparative study.

## 2. Methods

This was a retrospective, nonrandomized comparative case series (with the approval of the local Ethics Committee requiring no trial registration number). Medical records of consecutive patients, where either CP or HM implantation was uneventfully performed in one eye from January 2011 to January 2012 at the University Hospital of Parma (Italy), were reviewed. All surgeries were performed by two surgeons (Stefano A. Gandolfi and Nicola Ungaro), and all study subjects signed a dedicated informed consent prior to surgery. The series included subjects under local treatment for primary or secondary (e.g., pseudoexfoliative and pigmentary) open-angle glaucoma, referred to as the Glaucoma Service of our Institution Parma for uncontrolled IOP. The eyes were addressed to either one of the two procedures since, according to the EGS guidelines [10], the estimated postsurgery target IOP was arbitrarily set in the mid-to-high teens range by the two surgeons (Stefano A. Gandolfi and Nicola Ungaro).

The following data were collected from a total of 45 patients (45 eyes, 24 CP, and 21 HM) with a minimum follow-up of two years: (a) demographics, (b) IOP, (c) best corrected visual acuity (BCVA, tested by logarithmic chart at 4 metres), (d) Visual Field Mean Defect (MD, Humphrey 24-2 SITA-Standard program, Carl Zeiss Meditec Inc., Dublin, CA), (e) the number and type of hypotensive medications, (f) and the need for further glaucoma surgery. IOP was measured using Goldmann applanation tonometer and following the standard operating procedures (SOP) of the European Vision Clinical Research network (EVICR.net) which certified our Institution (certificate number: ECR37/2014), namely, (a) patients in sitting position at the slit lamp; (b) fluorescein staining with a standard fluorescein paper strip; (c) two rapidly consecutive readings, averaged with ≤2 mmHg IOP difference, with a third reading being performed when the difference was >2 mmHg.

The eyes were labelled as “complete” success, “qualified” success, or “failure” if, two years after surgery, they needed* no* hypotensive medications,* some* hypotensive medications, or further glaucoma surgery to attain the target IOP, respectively.

### 2.1. Operative Techniques

All surgeries were single operations, not combined with phacoemulsification.

CP was performed according to the standard fashion described in previous reports [11, 12]. Briefly, after conjunctival dissection at the 12 o'clock limbus, a 5 × 5 mm partial thickness (50%) scleral flap was dissected followed by a 4 × 4 mm inner scleral flap at 95% depth. The inner flap dissection was carried forward until the SC was unroofed. The dissection was then carried into the clear cornea to create a 0.3 mm Descemet's window; the inner scleral flap was then removed and the inner wall of the canal was peeled off together with trabecular meshwork, until percolation of aqueous humour was observed. A microcatheter (iTrack-250A, iScience Interventional, Inc., Menlo Park, CA) was then inserted 360° in the canal; a 10-0 prolene suture was tied to the tip of the catheter, and the catheter was retracted backward and, in order to achieve viscodilation of the canal, sodium hyaluronate 1.4% (Healon GV, Advanced Medical Optics, Inc., Santa Ana, CA) was simultaneously injected. The 10-0 prolene suture was finally tightly tied in a loop with a slip knot, to obtain an inward traction. The scleral flap was secured back to the sclera with 10-0 nylon sutures to create a water-tight closure. The conjunctiva was then sutured to the limbus with 8-0 absorbable sutures.

HM implantation was performed as follows: after a peribulbar injection of 5 mL of lidocaine, the patients were placed under the microscope and the head tilted to allow a clear view of the angle structures with a gonioprism. A 1.2–1.5 mm clear cornea incision was properly made to access the targeted site for microstent placement. HMW viscoelastic was introduced for chamber maintenance and an optimum view. The Hydrus delivery cannula was then inserted through the incision. The bevelled tip of the cannula was used to perforate the trabecular meshwork, and the microstent was implanted into Schlemm's canal by advancing the tracking wheel with the index finger, leaving 1-2 mm (the inlet segment) remaining in the AC. In one of the selected cases, the microstent had to be retracted and reinserted in a different location. Upon confirmation of position in the canal, the delivery system was withdrawn and viscoelastic was removed; the AC was inflated with balanced salt solution to achieve normal IOP.

### 2.2. Statistical Analysis

Statistical data were processed with the SPSS package (SPSS Inc. Released 2007; SPSS for Windows, Version 16.0. Chicago, SPSS Inc.). In detail, Student's *t*-test was for mean confrontation between groups, Chi-squared test was for double and triple enter contingency tables. For 2 × 2 contingency tables, Fischer's test was applied. Statistical significance was set at *p* < 0.05. The normal distribution of the analyzed data was stated by verifying for each parameter that means were “almost equal to” medians and asymmetric within ±2.

## 3. Results

Twenty-four eyes of 24 patients (16 men, age range: 34–66 years) who underwent CP and twenty-one eyes of 21 patients (15 men, age range: 37–69 years) who underwent HM implantation were included in the study. We considered all the patients who successfully completed surgery and the 2-year follow-up (therefore no loss to follow-up). No significant difference was found between the two groups with respect to demographics (age: *p* = 0.31; gender: *p* = 0.59; race: all Caucasians; right versus left eye *p* = 0.67). The diagnosis was as follows: *n* = 28 primary open-angle glaucoma (POAG; 16 CP and 12 HM); *n* = 15 pseudoexfoliation syndrome (PEX: 8 CP and 7 HM); and *n* = 2 pigmentary glaucoma (PG: 2 HM). At preoperative evaluation (baseline), no significant differences in the number of ongoing hypotensive active substances (*p* = 0.23) and in the number of eyes previously treated with argon laser trabeculoplasty/selective laser trabeculoplasty (AST/SLT) were detected between groups (*p* = 0.34).

**Figure 1:**
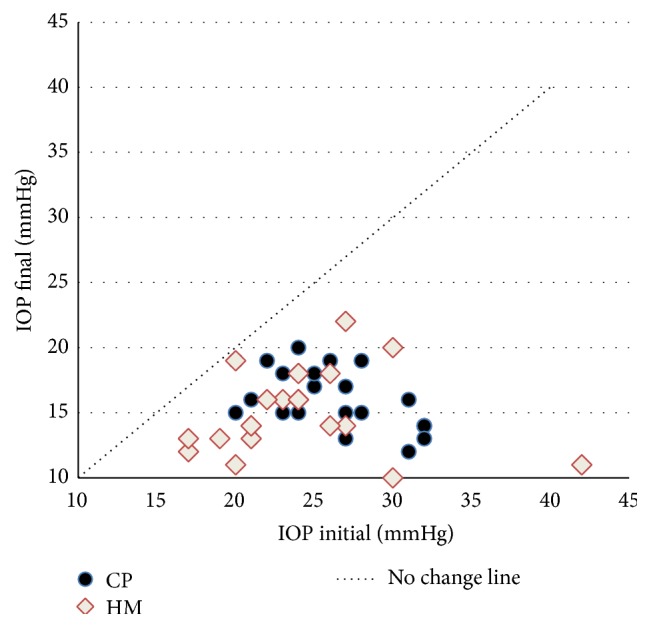
Scatterplot of preoperative (IOP initial) versus postoperative (IOP final) IOP values in the two treatment groups (CP = canaloplasty group; HM = Hydrus Microstent group).

Eye parameters at baseline and 2 years after surgery are detailed in Table 1 (mean ± SD). “IOP initial” refers to the IOP (on current hypotensive therapy) measured before surgery at the time of completion of the inpatient's record. “IOP final” refers to the IOP values measured 2 years after surgery (with a ±30-day time window), with or without medications. No intergroup difference was detected in either efficacy (IOP) or safety (i.e., BCVA and MD) outcomes. Conversely, an intragroup analysis showed that IOP significantly diminished in both groups upon surgery (*p* < 0.001). The IOP changes of individual eyes in each treatment group are shown in a scatterplot (Figure 1).  Table 2 shows the rate of each clinical outcome at the end of the follow-up. The distribution of the clinical success (“complete” or “qualified”) was not significantly different between the two groups (Pearson Chi-Square, *p* = 0.52; Likely Ratio, *p* = 0.51). Also the event “failure” (i.e., the need for further glaucoma surgery) was similar in the two groups either in terms of the number of procedures or the number of temporal latencies. Namely, two cases in the HM group were performed after 12 and 18 months of follow-up; two cases in the CP groups 12 and 13 months postoperatively.

**Figure 2:**
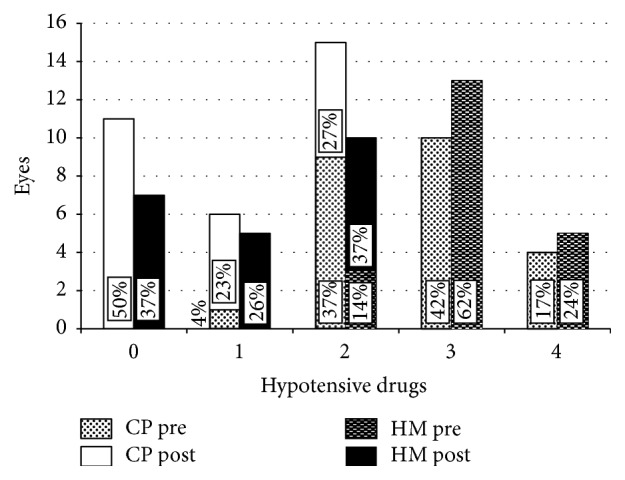
Distribution of eyes (eyes) referred to the number of required hypotensive medications (hypotensive drugs) in each treatment group, preoperatively and at the end of the 2-year follow-up. CP pre: preoperative distribution of eyes addressed to canaloplasty; CP post: distribution of eyes addressed to canaloplasty 24 months after surgery; HM pre: preoperative distribution of eyes addressed to Hydrus Microstent; HM post: distribution of eyes addressed to Hydrus Microstent 24 months after surgery.

Concerning the number of hypotensive active substances administered at the end of the follow-up, the mean values were in the CP group 0.7 ± 0.9 and in the HM group 0.9 ± 0.9. Differences referred to the intensity of the regimen (i.e., none, 1 or more active substances) are shown in Figure 2; no statistical difference was observed (Pearson Chi-Square = Likely Ratio; *p* = 0.74).

The final distribution among the subgroups of clinical outcome of the eyes previously treated by AST/SLT is displayed in Table 3. A laser treatment was paralleled by a lower rate of complete success in the CP group versus the HM group with borderline significance (Fisher exact test, *p* = 0.04). No effect of AST/SLT on the failure rate was observed instead.

As far as complications are concerned, a transient hyphema proved to be the most commonly described adverse event (7/24 eyes in the CP group and 4/21 eyes in the HM group). The hyphema cleared completely over few days in all the affected patients. An early postoperative IOP peak (≥30 mmHg within the first 48 hours) was recorded in 3 eyes in the CP group and in 1 eye after HM implantation. A YAG laser procedure was performed in 6 eyes in the CP group (goniopuncture) and in 4 eyes in the HM group (lysis of peripheral anterior synechiae) during follow-up.

## 4. Conclusion

An increasing interest in novel “blebless” antiglaucoma surgeries is documented worldwide. In particular, procedures aiming to restore the physiological outflow through the trabecular meshwork/SC complex are being developed. In the hereby presented study, we retrospectively compared the midterm clinical outcomes of CP, that is, an ab-externo approach to redilate the SC, versus an ab-interno procedure such as the implantation of a scaffold (HM).

Two years after surgery, both procedures were effective in decreasing IOP. The percentage of complete success in the CP group (50%) proved to be comparable with the 55% and 46% reported after a similar follow-up by Lewis and coworkers (2009) and Brusini (2014), respectively [13, 14]. Matlach and coworkers (2015) found a slightly lower rate of complete success (39%), but the two series are less directly comparable because of the lack of a cut-off IOP to define the postoperative success in our study [15]. Since the shortage of the so far published data, the results collected in our HM group cannot be valuably compared with prior series. A recent report showed the high rate of success (80%) of HM combined with cataract surgery [16]. If we had performed combined surgery, we would have probably achieved better results, too. However, since scaffolds aim to restore the outflow within the physiological range, the “midteens” IOP values observed postoperatively in our HM group are consistent with the expected mechanism of action of the procedure. Because of the retrospective nature of the study, no preset cut-off IOP values were planned. In fact, the target IOP was individually set by the surgeons according to what was suggested by the EGS guidelines for mild-to-moderate glaucoma damage with high starting IOP (i.e., a target IOP in the “mid-to-high teens”).

Comparing the two treatment groups, in our study the efficacy profile of CP was statistically comparable to that of HM. Both procedures are theoretically endowed within similar mechanisms of action. Therefore, our results are not at all surprising. A slightly (albeit not significant) better trend for clinical success in the CP group could be inferred by a qualitative evaluation of data in Table 2 and Figure 2: two years after surgery, 50% of eyes treated by CP maintained the target IOP without medications; 57% of HM implanted eyes required any medical treatment to attain similar IOP. A greater sample size, together with a longer follow-up, could potentially offer statistical significance to this observation. Also the event “failure” (i.e., the need for further glaucoma surgery) occurred similarly in the two groups, either in terms of number of cases (two in each treatment group) or number of temporal latencies. However, since CP is not a conjunctival-sparing procedure, a further limbal filtration surgery, if needed, is likely to be less successful and technically compelling. Conversely, the clear cornea approach gives the HM procedure a less invasive profile in terms of surgical impact on the eye. In fact, when further surgery was needed in our study eyes, a tube was implanted after CP, meanwhile a plain nonpenetrating deep sclerectomy was successfully performed after HM.

Both procedures offered a comparable low complication rate in our study. In the literature, CP was widely associated with a low rate of side effects, mostly when compared to filtering surgery. Albeit more recent, also MIGS have been associated with a good safety profile in the short term to midterm [17].

In conclusion, our retrospective comparison showed that both CP and HM were safe and effective blebless procedures in early-to-mid stage open-angle glaucoma. However, along with the limited sample size and follow-up, its retrospective nature is a major point of weakness for the present study. This entailed the lack of preset: (a) randomization procedure, (b) cut-off values for target IOP, and (c) intermediate follow-up time points. The administration of a survey to evaluate quality of life, as proposed by Klink and coauthors in a quite similar setting, could represent a further improvement to the present findings [18].

## What Was Known

- Glaucoma surgeries now include procedures aiming to restore the physiological outflow through the trabecular meshwork/SC complex. This latter in particular can be successfully redilated via either an ab-externo (the “canaloplasty”) or an ab-interno (the “scaffolds”) approach.
- Outcomes of canaloplasty are quite well described and compared to other filtering techniques; much less is reported concerning scaffolds.

## What This Paper Adds

Within the frame of this retrospective comparative study, one finds the following:

- two years after surgery, both CP and the HM were effective in decreasing IOP;
- both procedures offered a low complication rate;
- a slightly (albeit not significant) better trend for a “complete” clinical success for CP needs further confirmation.

## Figures and Tables

**Table 1:** Parameters considered for the study at the baseline (initial) and after a 24-month follow-up (final) for the two treatment groups.

Treatment groups	IOP initial (mmHg)	IOP final (mmHg)	BCVA initial (LogMAR)	BCVA final (LogMAR)	MD initial (dB)	MD final (dB)
HM	24 ± 6	15 ± 3	0.11 ± 0.08	0.08 ± 0.02	4.6 ± 1.9	4.2 ± 1.9
CP	26 ± 4	16 ± 2	0.11 ± 0.08	0.09 ± 0.08	4.0 ± 3.2	3.9 ± 3.3

Statistics	*p* = 0.22	*p* = 0.18	*p* = 0.93	*p* = 0.58	*p* = 0.45	*p* = 0.70

HM: Hydrus Microstent; CP: canaloplasty; IOP: intraocular pressure; mmHg: millimeters of mercury; BCVA: best correct visual acuity; LogMAR: logarithm of the minimum angle of resolution; MD: Visual Field Mean Defect; dB: decibel.

**Table 2:** Eyes' distribution according to the clinical outcome at the end of the follow-up.

	HM	CP	Total
Complete success			
Count	7	12	19
% within group	33.3%	50%	42.2%
Qualified success			
Count	12	10	22
% within group	57.1%	41.7%	48.9%
Failure			
Count	2	2	4
% within group	9.5%	8.3%	8.9%
Total			
Count	21	24	45
% within group	100%	100%	100%

HM: Hydrus Microstent; CP: canaloplasty.

**Table 3:** Distribution of eyes between the treatment groups according to a previous treatment (yes) by argon laser trabeculoplasty/selective laser trabeculoplasty (AST/SLT). Within brackets, the number eyes showing a complete clinical success (i.e., no medications) at the end of the follow-up.

Preoperative ALT/SLT	HM	CP	Total
Yes			
Count	10	14	24
	(4)	(3)	(7)

No			
Count	11	10	21
	(3)	(1)	(4)

Total			
Count	21	24	45
	(7)	(4)	(11)

HM: Hydrus Microstent; CP: canaloplasty.
